# The Central Role of the Interventional Radiologist in Advanced Therapies for Pulmonary Embolism: Results from An Online Member Survey by the Cardiovascular and Interventional Radiological Society of Europe

**DOI:** 10.1007/s00270-025-03998-9

**Published:** 2025-03-13

**Authors:** Gerard O’Sullivan, Stefan Müller-Hülsbeck, Patrick Haage, Florian Wolf, Mohamad Hamady, Birgit Slijepčević, Romaric Loffroy, Fabrizio Fanelli, Hicham Kobeiter, Robert A. Morgan

**Affiliations:** 1 University Hospital Galway, Galway, Ireland; 2 Academic Hospital Christian-Albrechts-University Kiel, Kiel, Germany; 3 Helios University Hospital, University Witten/Herdecke, Wuppertal, Germany; 4 Division of Cardiovascular and Interventional Radiology, Medical University of Vienna, Vienna, Austria; 5 Imperial College, St Mary’s Campus, London, UK; 6 Cardiovascular and Interventional Radiological Society of Europe, Vienna, Austria; 7 Department of Diagnostic and Interventional Radiology, François-Mitterrand University Hospital, Dijon, France; 8 Vascular and Interventional Radiology Department, “Careggi” University Hospital – University of Florence, Florence, Italy; 9 Radiology Department, H. Mondor Hospital, Assistance Publique-Hôpitaux de Paris, University Paris Est Creteil, Creteil, France; 10 St George’s University of London, London, UK

**Keywords:** Current practice, Member survey, Pulmonary embolism, Catheter-directed thrombolysis, Thrombectomy

## Abstract

**Purpose:**

To describe the outcomes of a survey on the provision of interventional radiology procedures for the treatment of acute pulmonary embolism (PE) in Europe and beyond.

**Methods:**

An online survey with 14 structured items was designed by the authors and was sent to 7116 CIRSE members via email. The anonymous online survey collected data for eight weeks; only complete responses were statistically analysed.

**Results:**

The survey was completed by 373 members (5.24%). Among these, 75.1% worked at centres offering catheter-directed thrombolysis or thrombectomy, in which 89.3% (250) personally perform endovascular treatment techniques for pulmonary embolism and the IR department is primarily responsible for the endovascular treatment techniques of PE in 83.2% of cases. The most frequently used endovascular techniques were (large bore) aspiration thrombectomy (85%) and catheter-directed thrombolysis (58.9%). The most common indications for intervention were sub-massive and massive PE (69.9%) and massive PE only (28%). In 70% of centres offering catheter-directed thrombolysis or thrombectomy, three or more Interventional Radiologists (IRs) are involved in PE treatment. Multidisciplinary rapid response teams for PE were available in 40.8% of centres, and included IRs in 91.4%.

**Conclusion:**

IRs are heavily involved in the management of patients with massive and sub-massive pulmonary embolism; further research is mandated to address clinical questions including patient selection and the timing for transcatheter therapies of PE provided by IR.

**Graphical Abstract:**

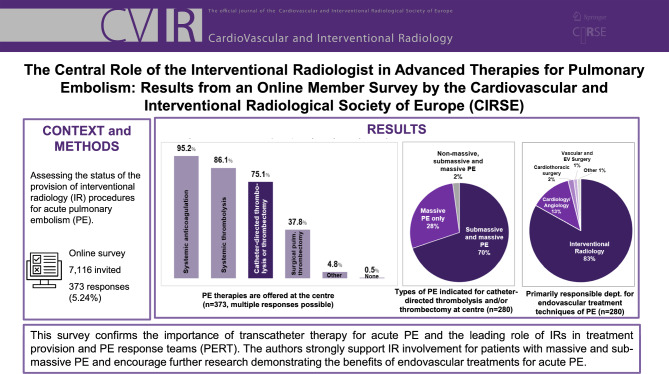

**Supplementary Information:**

The online version contains supplementary material available at 10.1007/s00270-025-03998-9.

## Introduction

Pulmonary embolism (PE) is a major health issue and is an area where interventional radiologists (IRs) can play a crucial role in emergency situations [1–4]. CIRSE’s Endovascular Subcommittee identified this topic as a current priority and surveyed CIRSE members on the provision of interventional radiology procedures for the treatment of acute PE. This manuscript reports the outcomes of that survey.

## Materials and Methods

A questionnaire consisting of 10 single-choice questions, three multiple choice questions and one open-text question, was devised by the authors. The survey was programmed in an online survey tool (Alchemer LLC, USA) and included display logic for follow-up questions based on previous responses given, to make the survey as intuitive and practical for responders as possible. Following three initial questions regarding general demographics, a question on the PE therapies offered at the respective centre split the responders into different sub-sets with more detailed follow-up questions offered to those who selected “catheter-directed thrombolysis or thrombectomy” as a treatment option available at their centre. At the end of the survey, all responders were asked to respond to four general questions about the perceived safety and status of endovascular treatment options for PE, as well as tools that could help them in their daily practice and their awareness of the European Certification for Endovascular Specialists.

A total of 7116 CIRSE members were invited via email to take the survey on January 25, 2024. The survey was a completely anonymous online questionnaire. Two reminders were sent, and the survey was closed on March 22, 2024. All complete responses were statistically analysed in Microsoft Excel 365 (2024, Microsoft Corporation, USA) by the authors, using descriptive statistical analysis.

## Results

The survey yielded a total of 373 complete responses and a response rate of 5.24%. Among CIRSE members who are certified as endovascular experts through the European Board of Interventional Radiology—Endovascular Specialist diploma (EBIR-ES), the response rate was considerably higher, with 75% of all EBIR-ES holders (*n* = 51) having answered the survey. European responders represented 74.3% of the sample, with the highest number of responses collected from IRs based in Italy (11.3%), Germany (11%), the United Kingdom (8.8%), Spain (7.2%) and Australia (5.9%). Similar to previous CIRSE surveys, the majority (59.5%) worked in teaching or university hospitals, 29.2% in general or public hospitals and 10.7% in private clinics or hospitals.

**Fig. 1:**
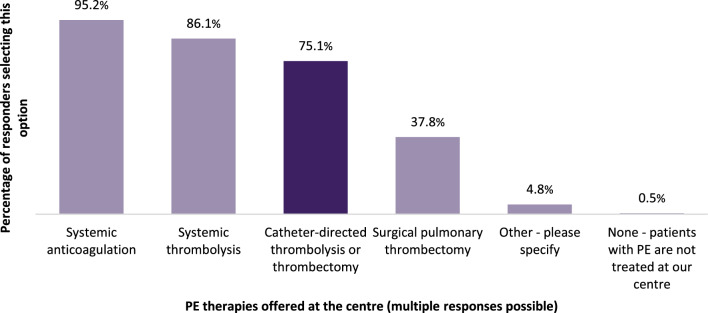
PE therapies offered at the responders’ centres; multiple responses possible

All survey responders (373) were asked to indicate the PE therapies offered at their respective centres. Figure 1 depicts the PE therapies offered at responders’ centres. For “other” therapies, responders most frequently indicated extracorporeal membrane oxygenation (ECMO, 9 counts).

**Fig. 2:**
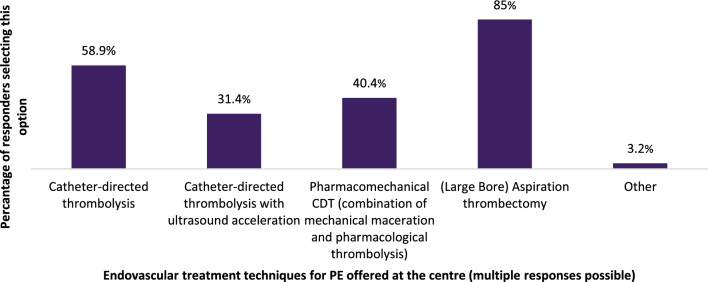
Endovascular treatment techniques for PE responders’ centres; multiple responses possible

Responders who indicated that catheter-directed thrombolysis or thrombectomy were offered at their centre (*n* = 280) were asked about the endovascular treatment techniques of PE used at their centres (Fig. 2). The most frequently indicated technique was (large bore) aspiration thrombectomy (85%), followed by catheter-directed thrombolysis (58.9%), pharmaco-mechanical CDT (combination of mechanically maceration and pharmacological thrombolysis, 40.4%) and catheter-directed thrombolysis with ultrasound acceleration (31.4%).

**Fig. 3:**
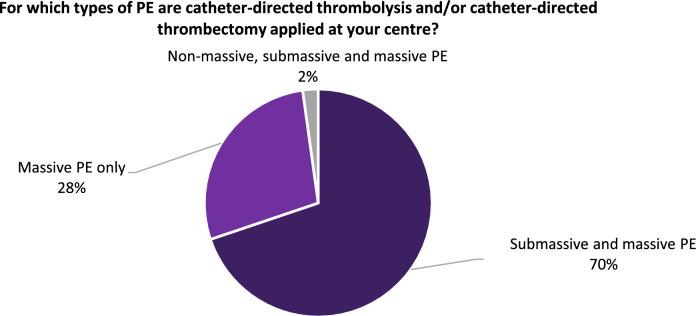
Types of PE for which catheter-directed thrombolysis and/or catheter-directed thrombectomy applied

A large majority of responders in this group (69.9%) specified that catheter-directed thrombolysis and/or catheter-directed thrombectomy were utilised for sub-massive and massive PE [4], 28% replied that these are used for massive PE only and 2.2% replied that these are used for non-massive, sub-massive and massive PE (see Fig. 3).

**Fig. 4:**
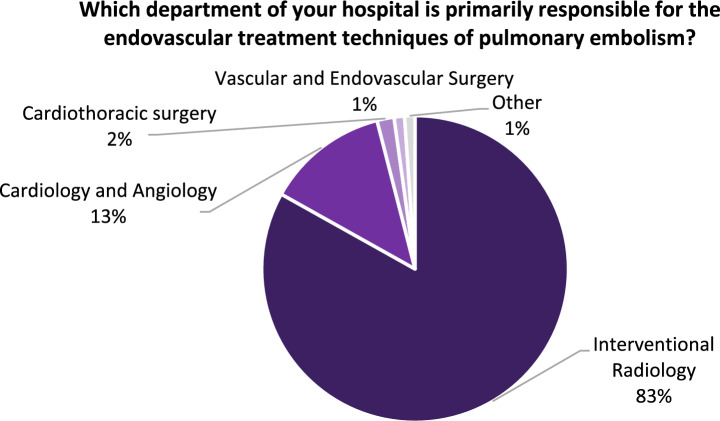
Departments primarily responsible for endovascular treatment techniques of PE

Among all responders working in centres where catheter-directed thrombolysis or thrombectomy were offered (*n* = 280), 89.3% (*n* = 250) personally performed endovascular treatment techniques for PE; with the IR department being primarily responsible for the endovascular treatment techniques of PE in 83.2% of cases, followed by the cardiology and angiology departments (12.9%), cardiothoracic surgery (1.8%), vascular and endovascular surgery (1.1%), and other departments (1.1%) (see Fig. 4). Finally, this subsample was asked how many IR colleagues can perform endovascular techniques of PE, with the options of 5 or more colleagues (29.6%), 4 colleagues (20.7%), 3 colleagues (18.9%), 2 colleagues (17.9%), 1 colleague (8.2%), or no other colleague (4.6%).

All responders were asked whether a multidisciplinary rapid response team model, Pulmonary Embolism Response Team (PERT), was implemented at their centre, which was the case for 40.8% (*n* = 152). The disciplines involved in the PERT team (*n* = 152, multiple responses possible) included interventional radiologists (91.4%), critical care and emergency medicine physicians (87.5%), cardiologists (69.7%), pulmonologists (55.9%) and cardiothoracic surgeons (42.1%), as well as other disciplines (12.5%).

In their daily practice and/or derived from literature, 85.8% of responders considered the endovascular treatment of PE as safe, 12.6% were undecided and 1.6% answered negatively. Judging from available literature and practice in their departments, 49.2% considered the use of endovascular treatment options as the primary therapy regimen in sub-massive and massive PE after evaluation in the multidisciplinary team; 29.6% agreed with endovascular treatment as primary therapy regimen if there is an absolute contraindication for systemic thrombolysis, a failure of systemic thrombolysis, or no time for the 2 h of administration of systemic thrombolytic agents; 9.4% agreed with the endovascular treatment as primary therapy regimen no matter what, while 6.2% remained undecided and 5.6% responded negatively due to the lack of multicentre, randomised controlled trials.

## Discussion

The survey was answered by a relatively specialised sample of interventional radiologists, with a majority of responders personally performing endovascular treatment techniques for PE, and 44% being holders of the European Certification for Endovascular Specialists (EBIR-ES) or planning to get certified.

While systemic anticoagulation remains the gold standard treatment for PE, three quarters of centres in the present survey offer catheter-directed thrombolysis or thrombectomy. Among endovascular treatment techniques of PE in these centres, the key modalities are (large bore) aspiration thrombectomy (85%), followed by catheter-directed thrombolysis (58.9%).

Importantly, the survey also shows that, in the present sample, IRs are leading the delivery of catheter-based therapies for PE, with 83.2% confirming that the IR/Radiology department leads the provision of these services. IRs have a strong presence in PERT teams (91.4%) whenever these are available. In 70% of centres, the provision of endovascular treatments for PE treatments is ensured by three or more IRs, which is an encouraging number regarding staffing.

Endovascular treatment options for PE are also considered as safe by a large majority (85.8%), and the therapy of choice for sub-massive and massive PE. The authors feel that this strong support for endovascular therapies for PE needs to translate into further recognition of the importance of these modalities provided by IRs, with the support of high-quality clinical studies.

The results from this survey highlight the need for increasing promotion of transcatheter therapy for significant PE and the role of IRs in these therapies. There is also a pressing need for guidelines or standards of practice documents, and data from randomised controlled trials, registry and cohort studies to confirm the benefit of endovascular interventions for patients with severe PE.

Regarding the limitations of the results of this survey, a selection bias towards responders with an interest or strong opinion on the topic must be acknowledged, and while CIRSE with almost 10,000 members can be considered as representative of the European IR community, the present sample is relatively small. Another limitation may be that the actual size of large bore thrombectomy devices was not defined for the survey.

## Conclusion

This survey has shown the importance of transcatheter therapy for acute PE and has confirmed that IRs play a leading role in the provision of endovascular treatment techniques for PE as well as being key members of Pulmonary Embolus Response Teams (PERT).

The authors strongly support that IRs must be involved in the treatment of patients with massive and sub-massive PE. Further research to demonstrate the benefits of PE therapy and to further specify the most suitable patients for transcatheter therapy for acute PE are required.

## Supplementary Information

Below is the link to the electronic supplementary material.

Supplementary file1 (DOCX 17 kb)
