# Supplementary material for: The Central Role of the Interventional Radiologist in Advanced Therapies for Pulmonary Embolism: Results from An Online Member Survey by the Cardiovascular and Interventional Radiological Society of Europe
Source: Cardiovasc Intervent Radiol. 2025 Mar 13;48(6):851–6. doi: 10.1007/s00270-025-03998-9 (PMC12170684; doi:10.1007/s00270-025-03998-9)
Supplement: Supplementary file 1 — Supplementary file1 (DOCX 17 kb) [file 270_2025_3998_MOESM1_ESM.docx]

**For the Online supplement:**

# CIRSE Member Survey on Advanced Therapies for Pulmonary Embolism – List of questions

#### 1) Where is your centre located?

[Country dropdown]

#### 2) What type of centre do you work in?

( ) General/public hospital

( ) Teaching/University hospital

( ) Private hospital/clinic, foundation, etc.

( ) Other (please specify):: _________________________________________________

#### 3) Which of the following PE therapies are offered at your centre? Please select all that apply:

[ ] Systemic anticoagulation

[ ] Systemic thrombolysis

[ ] Catheter-directed thrombolysis or thrombectomy

[ ] Surgical pulmonary thrombectomy

[ ] Other - please specify: _________________________________________________

[ ] None - patients with PE are not treated at our centre

#### 4) Which of the following endovascular treatment techniques of pulmonary embolism are used at your centre?

#### *[only displayed if #3 question "Which of the following PE therapies are offered at your centre?” is answered by "Catheter-directed thrombolysis or thrombectomy".]*

#### Please select all that apply:

[ ] Catheter-directed thrombolysis

[ ] Catheter-directed thrombolysis with ultrasound-acceleration

[ ] Pharmacomechanical CDT (combination of mechanically maceration and pharmacological thrombolysis)

[ ] Aspiration thrombectomy

[ ] Other - please specify: _________________________________________________

#### 5) For which types of PE are catheter-directed thrombolysis and/or catheter-directed thrombectomy applied at your centre?

#### *[only displayed if #3 question "Which of the following PE therapies are offered at your centre?” is answered by "Catheter-directed thrombolysis or thrombectomy".]*

( ) Non-massive, submassive and massive PE

( ) Submassive and massive PE

( ) Massive PE only

#### 6) Is there a multidisciplinary rapid response team model Pulmonary Embolism Response Team (PERT) implemented at your centre?

#### *[only displayed if #3 question "Which of the following PE therapies are offered at your centre?” is answered by either of the following: "Systemic anticoagulation","Systemic thrombolysis","Catheter-directed thrombolysis or thrombectomy","Surgical pulmonary thrombectomy","Other - please specify"]*

( ) Yes

( ) No

#### 7) Which disciplines are involved in the Pulmonary Embolism Response Team (PERT) at your centre?

#### *[only displayed if #6 question “*Is there a multidisciplinary rapid response team model Pulmonary Embolism Response Team (PERT) implemented at your centre?” is answered “Yes”]

[ ] Pulmonologists

[ ] Cardiologists

[ ] Interventional Radiologists

[ ] Cardiothoracic surgeons

[ ] Critical care and emergency medicine physicians

[ ] Other - please specify: _________________________________________________

#### 8) Do you personally perform endovascular treatment techniques for pulmonary embolism?

#### *[only displayed if #3 question "Which of the following PE therapies are offered at your centre?” is answered by "Catheter-directed thrombolysis or thrombectomy".]*

( ) Yes

( ) No

#### 9) Which department of your hospital is primarily responsible for the endovascular treatment techniques of pulmonary embolism?

#### *[only displayed if #3 question "Which of the following PE therapies are offered at your centre?” is answered by "Catheter-directed thrombolysis or thrombectomy".]*

#### Please select all that apply:

( ) Interventional Radiology

( ) Cardiology and Angiology

( ) Vascular and Endovascular Surgery

( ) Cardiothoracic surgery

( ) Other - please specify: _________________________________________________

#### 10) How many IR colleagues can perform endovascular treatment techniques of pulmonary embolism at your centre?

( ) 0

( ) 1

( ) 2

( ) 3

( ) 4

( ) 5 or more

#### 11) In your daily practice and/or derived from literature, do you consider the endovascular treatment of PE as safe?

( ) Yes

( ) No

( ) I am undecided

#### 12) Judging from available literature and practice in your department, do you consider the use of endovascular treatment options as the primary therapy regimen in submassive and massive PE?

( ) Yes, if there is an absolute contraindication for systemic thrombolysis, a failure of systemic thrombolysis or no time for the 2 h of administration of systemic thrombolytic agents

( ) Yes, no matter what, it has to be implemented as a primary therapy to avoid secondary adverse events due to thrombolytics

( ) No, as long as it is not recommended in guidelines

( ) No, since multicentre, randomized controlled trials are lacking

( ) I am undecided

### 13) Generally speaking, which tools / documents / guidelines that are not yet available would help you in your daily endovascular work?

[open text]

#### 14) Are you aware of the [European Certification for Endovascular Specialists (EBIR-ES)](https://www.cirse.org/certification/certification-endovascular-specialists/) offered by CIRSE?

( ) Yes, I am already certified

( ) Yes, I am planning to get certified

( ) Yes, but it is not relevant for me

( ) No, I have never heard of this certification

( ) No, but I would be interested to learn more
